# Supplementary material for: A scoping review of “Tang ping” (Lying flat) and mental health status among Chinese youth
Source: PLoS One. 2026 Mar 10;21(3):e0342591. doi: 10.1371/journal.pone.0342591 (PMC12974857; doi:10.1371/journal.pone.0342591)
Supplement: S1 File — (PDF) [file pone.0342591.s001.pdf]

Preferred Reporting Items for Systematic reviews and Meta-Analyses extension for Scoping Reviews (PRISMA-ScR) Checklist

SECTION	ITEM	PRISMA-ScR CHECKLIST ITEM	REPORTED ON PAGE #
**TITLE**			
Title	1	Identify the report as a scoping review.	A Scoping Revi
**ABSTRACT**			
Structured summary	2	Provide a structured summary that includes (as applicable): background, objectives, eligibility criteria, sources of evidence, charting methods, results, and conclusions that relate to the review questions and objectives.	tal health status presented by "Tang ping" students and young workers.
**INTRODUCTION**			
Rationale	3	Describe the rationale for the review in the context of what is already known. Explain why the review questions/objectives lend themselves to a scoping review approach.	vidence to help youth improve their "Tang ping" behavior.
Objectives	4	Provide an explicit statement of the questions and objectives being addressed with reference to their key elements (e.g., population or participants, concepts, and context) or other relevant key elements used to conceptualize the review questions and/or objectives.	s, "Tang ping" (I), and the context is mental health status (Co).
**METHODS**			
Protocol and registration	5	Indicate whether a review protocol exists; state if and where it can be accessed (e.g., a Web address); and if available, provide registration information, including the registration number.	https://www.acpjournals.org/doi/10.7326/M18-0850
Eligibility criteria	6	Specify characteristics of the sources of evidence used as eligibility criteria (e.g., years considered, language, and publication status), and provide a rationale.	See in Table 2.
Information sources*	7	Describe all information sources in the search (e.g., databases with dates of coverage and contact with authors to identify additional sources), as well as the date the most recent search was executed.	See in Table 2. Searched from Feb.3rd to Mar. 21st.
Search	8	Present the full electronic search strategy for at least 1 database, including any limits used, such that it could be repeated.	See in Table 1.
Selection of sources of evidence†	9	State the process for selecting sources of evidence (i.e., screening and eligibility) included in the scoping review.	articles by scoping review.
Data charting process‡	10	Describe the methods of charting data from the included sources of evidence (e.g., calibrated forms or forms that have been tested by the team before their use, and whether data charting was done independently or in duplicate) and any processes for obtaining and confirming data from investigators.	nd the process was completed independently and reproducibly and approved by all authors.
Data items	11	List and define all variables for which data were sought and any assumptions and simplifications made.	
Critical appraisal of individual sources of evidence§	12	If done, provide a rationale for conducting a critical appraisal of included sources of evidence; describe the methods used and how this information was used in any data synthesis (if appropriate).	quality assessment of the included literature is performed.
Synthesis of results	13	Describe the methods of handling and summarizing the data that were charted.	ble 3 and Figs 2-6.

Table 2 Inclusion and Exclusion Criteria

Feature	Inclusion Criteria	Exclusion Criteria
Study Design	Quantitative or qualitative or mixed method papers on "Tang ping" among Chinese youth's mental health	Papers that do not focus on Chinese youth or "Tang ping" behaviour or mental health status
Participants	Chinese youth	Papers that do not include Chinese youth
Outcomes	Reports data findings on "Tang ping"	Papers that do not report relevant outcomes
Time	From 2021 until 2024	Outside of 2021-2024
Language	English and Chinese	Papers in other languages
Information Sources	CNKI, Google Scholar, Scopus, and Elicit (supplement)	Papers are not in CNKI, Google Scholar, or Scopus

Table [illegible]

Chines[illegible]

Filter c[illegible]

Step				
#1	CNKI	"Tang ping" AND (College students OR youth OR young people) AND "Mental health"	Title/Abstract/ Keywords	352
#2	Scopus	"Tang ping" OR "lying flat"	Title/Abstract/ Keywords	15
#3	Google Scholar	("Tang ping" OR "lying flat") AND (College students OR youth OR young people) AND(Mental health OR psychological problem OR psychological well-being OR depression OR anxiety OR burnout OR academic problems OR Internet addiction)	Title/Abstract/ Keywords	598
#4	Elicit (Additional literature search)	"lying flat" AND "college students" AND "mental health"		8

Variables	Definition	Assumptions and Simplifications
Publication Type	Articles in Journal	——
Publication Year	2021-2024	4 years
Tang ping	Four types of definition	There are different types and classifications
Mental health status	The mental health status related to "Tang ping" can be divided into negative affect and positive affect	Negative affects are more than positive affects in mental health among "Tang ping" youth

St. Michael's
Inspired Care.
Inspiring Science.

SECTION	ITEM	PRISMA-ScR CHECKLIST ITEM	REPORTED ON PAGE #
**RESULTS**			
Selection of sources of evidence	14	Give numbers of sources of evidence screened, assessed for eligibility, and included in the review, with reasons for exclusions at each stage, ideally using a flow diagram.	See in Fig.1
Characteristics of sources of evidence	15	For each source of evidence, present characteristics for which data were charted and provide the citations.	See in excel-PRISMA-ScR17.
Critical appraisal within sources of evidence	16	If done, present data on critical appraisal of included sources of evidence (see item 12).	[illegible] included literature is performed.
Results of individual sources of evidence	17	For each included source of evidence, present the relevant data that were charted that relate to the review questions and objectives.	See in excelPRISMA-ScR17.
Synthesis of results	18	Summarize and/or present the charting results as they relate to the review questions and objectives.	young workers.
**DISCUSSION**			
Summary of evidence	19	Summarize the main results (including an overview of concepts, themes, and types of evidence available), link to the review questions and objectives, and consider the relevance to key groups.	P30,L614, In conclusion part: The results showed tha [illegible]
Limitations	20	Discuss the limitations of the scoping review process.	P29,L600-608,F
Conclusions	21	Provide a general interpretation of the results with respect to the review questions and objectives, as well as potential implications and/or next steps.	ns for "Tang ping" Chinese youth.
**FUNDING**			
Funding	22	Describe sources of funding for the included sources of evidence, as well as sources of funding for the scoping review. Describe the role of the funders of the scoping review.	———————

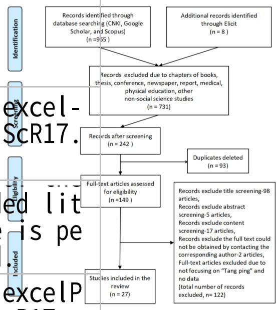

JBI = Joanna Briggs Institute; PRISMA-ScR = Preferred Reporting Items for Systematic reviews and Meta-Analyses extension for Scoping Reviews.

* Where *sources of evidence* (see second footnote) are compiled from, such as bibliographic databases, social media platforms, and Web sites.

† A more inclusive/heterogeneous term used to account for the different types of evidence or data sources (e.g., quantitative and/or qualitative research, expert opinion, and policy documents) that may be eligible in a scoping review as opposed to only studies. This is not to be confused with *information sources* (see first footnote).

‡ The frameworks by Arksey and O'Malley (6) and Levac and colleagues (7) and the JBI guidance (4, 5) refer to the process of data extraction in a scoping review as data charting.

§ The process of systematically examining research evidence to assess its validity, results, and relevance before using it to inform a decision. This term is used for items 12 and 19 instead of "risk of bias" (which is more applicable to systematic reviews of interventions) to include and acknowledge the various sources of evidence that may be used in a scoping review (e.g., quantitative and/or qualitative research, expert opinion, and policy document).

From: Tricco AC, Lillie E, Zarin W, O'Brien KK, Colquhoun H, Levac D, et al. PRISMA Extension for Scoping Reviews (PRISMAScR): Checklist and Explanation. Ann Intern Med. 2018;169:467–473. doi: 10.7326/M18-0850.
