# Supplementary material for: A scoping review of “Tang ping” (Lying flat) and mental health status among Chinese youth
Source: PLoS One. 2026 Mar 10;21(3):e0342591. doi: 10.1371/journal.pone.0342591 (PMC12974857; doi:10.1371/journal.pone.0342591)
Supplement: Attachment — Submitted filename: PLOS ONE Reviewer Comments #.docx [file pone.0342591.s003.docx]

**PLOS ONE Reviewer Comments**

**1. Validity of the Study**
The manuscript follows a scoping review methodology and refers to PRISMA-ScR, which is appropriate for the topic. The inclusion of CNKI ensures coverage of Chinese-language studies, which is important. However, the search was limited to three main databases, which may restrict its comprehensiveness. The absence of a quality assessment is acknowledged, but a clearer justification strengthens validity. Overall, the study is methodologically sound, although transparency in reporting could be improved in the Methods and Results sections.

**2. Originality and Contribution to the Field**
The topic of “Tang Tang is highly original and culturally significant. To my knowledge, this is the first scoping review to synthesize evidence on “Tang ping” and mental health status in Chinese youth. This paper brings together different conceptualizations of “Tang ping” and links them to both negative and positive mental health outcomes. This study makes a meaningful contribution to youth studies, psychology, and cross-cultural social sciences.

**3. Quality of Presentation**
The manuscript is generally well structured but can benefit from language editing to improve clarity and readability. The results section is somewhat lengthy and repetitive; summarizing findings in tables or diagrams (e.g., categorization of “Tang ping” types, positive/negative mental health outcomes) would enhance accessibility. The figures and tables in the current draft appear incomplete or not fully formatted and should be revised before publication.

**4. Importance of Findings to the Community**
The findings are relevant not only for Chinese social and mental health research but also for understanding broader global youth responses to social pressure and overwork. This study has implications for policymakers, educators, and health professionals concerned with youth well-being. It also highlights the need for more longitudinal and interventional research, which is an important message for the scholarly community.

**5. Specific Comments for Improvement**

- Clarify the primary research question(s) in the Introduction to ensure consistency.
- We expanded slightly on why databases beyond CNKI, Scopus, and Google Scholar were not included.
- Provide justification for the decision to not conduct a formal quality assessment of the included studies.
- Consider reorganizing the results to avoid repetition, possibly using summary tables or schematic diagrams.
- The abstract should be shortened to emphasize key findings and implications, rather than methodological details.
- Revise figures and tables to ensure clarity, captions, and correct formatting.
- A light copyedit to smooth grammar and simplify long sentences would improve the readability.

**Overall Recommendation:**
This valuable and timely study. Revisions focused on presentation and clarity could make a strong contribution to PLOS ONE. I recommend **minor to moderate revision**.
